# Bronchiectasis as the Sentinel Manifestation of ANCA‐Associated Vasculitis: A Case Report and Literature Review

**DOI:** 10.1002/rcr2.70777

**Published:** 2026-10-01

**Authors:** Andrew Kim, Celine Vartany, Siddhanth Hegde, Kristie Lau, Lynette Tatosyan, Brannen Liang

**Affiliations:** ^1^ Department of Internal Medicine Kaiser Permanente Los Angeles Medical Center Los Angeles California USA; ^2^ Department of Radiology Kaiser Permanente Los Angeles Medical Center Los Angeles California USA; ^3^ Department of Rheumatology Kaiser Permanente Los Angeles Medical Center Los Angeles California USA; ^4^ Department of Pulmonary and Critical Care Medicine Kaiser Permanente Los Angeles Medical Center Los Angeles California USA

**Keywords:** ANCA‐associated vasculitis, bronchiectasis, crescentic glomerulonephritis, diagnostic delay, MPO‐ANCA

## Abstract

Bronchiectasis in ANCA‐associated vasculitis (AAV) is usually attributed to structural damage from established disease. We report a 74‐year‐old woman with 7 years of culture‐negative bronchiectasis, incompletely responsive to corticosteroids, in whom AAV was diagnosed after she developed palpable purpura, haemoptysis, hematuria and acute kidney injury. MPO‐ANCA was markedly elevated (250.2 U/mL; reference < 1.0 U/mL) with PR3‐ANCA negative. Renal biopsy showed pauci‐immune crescentic glomerulonephritis, Berden focal class (one of 14 glomeruli with a cellular crescent). Induction with intravenous methylprednisolone and rituximab produced substantial renal recovery (eGFR nadir 23, stabilizing at 60 mL/min/1.73 m^2^ at 15 months) and near‐complete resolution of the pulmonary consolidations. This case describes an airway‐first AAV phenotype. She met none of the trigger features that current guidelines use to prompt autoimmune screening, identifying a diagnostic blind spot in progressive culture‐negative bronchiectasis without systemic features.

## Graphical abstract

A 74‐year‐old woman was treated for 7 years as culture‐negative bronchiectasis before palpable purpura, haemoptysis, and acute kidney injury revealed MPO‐ANCA vasculitis with pauci‐immune crescentic glomerulonephritis. Rituximab‐based induction produced near‐complete resolution of the pulmonary consolidations and sustained clinical remission at 15 months. Bronchiectasis may be the sentinel manifestation of ANCA‐associated vasculitis, yet it sits outside the trigger features that current guideline algorithms use to prompt ANCA testing.

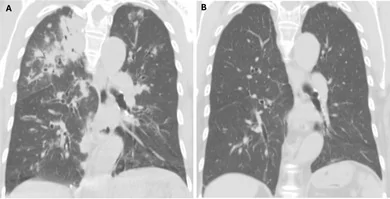

## 1 Introduction

ANCA‐associated vasculitis (AAV)—encompassing microscopic polyangiitis (MPA), granulomatosis with polyangiitis, and eosinophilic granulomatosis with polyangiitis (EGPA)—is a necrotizing small‐vessel vasculitis defined by pathogenic antineutrophil cytoplasmic antibodies [1]. Classical presentations prompt early immunological evaluation. Bronchiectasis is rarely regarded as an inaugural manifestation; when present it is usually attributed to structural damage from established vasculitic inflammation, a framework that can defer serological testing until systemic organ involvement declares itself. We describe a patient in whom bronchiectasis preceded systemic AAV by 7 years.

## 2 Case Report

A 74‐year‐old woman with intermittent asthma was evaluated in 2018 for chronic productive cough. High‐resolution CT (HRCT) showed focal right middle lobe bronchiectasis. Serial sputum cultures and bronchoalveolar lavage were negative for bacterial, mycobacterial and fungal pathogens, and non‐tuberculous mycobacterial infection, cystic fibrosis, primary immunodeficiency, EGPA, and allergic bronchopulmonary aspergillosis were excluded; spirometry was normal. Empiric corticosteroids and macrolides were given for presumed non‐infective airway inflammation; symptoms improved but never resolved. Over 6 years HRCT showed progression to multifocal bilateral bronchiectasis with tree‐in‐bud nodularity and mucus plugging. Antinuclear antibody was negative; ANCA serology was not obtained, and the diagnosis remained bronchiectasis of undetermined aetiology for 7 years (Table 1).

**TABLE 1:** Timeline of clinical, laboratory, and radiological findings from airway‐predominant disease to systemic ANCA‐associated vasculitis and treatment response.

Period	Clinical	Investigations	Imaging/histology
2018	Chronic productive cough	Sputum and BAL cultures negative (bacterial, mycobacterial, fungal); NTM, cystic fibrosis, immunodeficiency and ABPA excluded; spirometry normal	Focal right middle lobe bronchiectasis
2019–2024	Recurrent exacerbations; partial symptomatic response to corticosteroids and macrolides without resolution	Repeat cultures negative; ANA negative; ANCA not obtained. Total serum IgE 114 kU/L (2024); peripheral eosinophil counts normal throughout; FeNO not available.	Progression to multifocal bilateral bronchiectasis, tree‐in‐bud nodularity, mucus plugging and new parenchymal nodules
2025	Palpable purpura, haemoptysis, haematuria, AKI (non‐oliguric); haemoglobin and oxygen saturation stable	Creatinine 2.09 mg/dL (eGFR 24), from 0.46 mg/dL (eGFR 100) 6 weeks earlier; nadir 2.19 mg/dL (eGFR 23) day 3; p‐ANCA on IIF; MPO‐ANCA 250.2 U/mL (ref < 1.0), PR3‐ANCA negative; peripheral eosinophils 0%, 2+ proteinuria, dysmorphic RBCs, no casts; anti‐GBM, anti‐dsDNA negative, complement normal	Multifocal bilateral consolidations on established bronchiectasis, no DAH pattern (Figure 1A); renal biopsy: pauci‐immune crescentic GN, 1/14 glomeruli with cellular crescent, Berden focal class, no comment on eosinophilic infiltration
Induction	IV methylprednisolone 500 mg daily ×3, oral prednisone taper; rituximab 1 g repeated after 2 weeks, then 1 g every 6 months as maintenance; PJP prophylaxis	No infusion reactions	—
2 months	Asymptomatic	Peak eGFR 74 mL/min/1.73 m^2^	—
5 months	Asymptomatic	—	Near‐complete resolution of consolidations; bronchiectasis persists (Figure 1B)
12 months	Asymptomatic	MPO‐ANCA 5.2 U/mL; B‐cell depletion confirmed; IIF weakly positive	—
15 months	Asymptomatic; symptoms better controlled than in preceding 7 years	eGFR 60 mL/min/1.73 m^2^ (stage 3a CKD); no infections or corticosteroid complications	—

Abbreviations: AAV, ANCA‐associated vasculitis; ABPA, allergic bronchopulmonary aspergillosis; AKI, acute kidney injury; ANA, antinuclear antibody; ANCA, antineutrophil cytoplasmic antibody; BAL, bronchoalveolar lavage; CKD, chronic kidney disease; DAH, diffuse alveolar haemorrhage; eGFR, estimated glomerular filtration rate (CKD‐EPI 2021, mL/min/1.73 m^2^); FeNO, fractional exhaled nitric oxide; GBM, glomerular basement membrane; GN, glomerulonephritis; IgE, immunoglobulin E; IIF, indirect immunofluorescence; IV, intravenous; MPO, myeloperoxidase; NTM, non‐tuberculous mycobacteria; PJP, Pneumocystis jirovecii pneumonia; PR3, proteinase 3; RBC, red blood cell.

In 2025 she presented with palpable purpura of both lower extremities, haemoptysis, and hematuria. HRCT showed new multifocal bilateral consolidations superimposed on the established bronchiectasis (Figure 1A). Haemoglobin and oxygen saturation remained stable with no ground‐glass pattern of diffuse alveolar haemorrhage; bronchoscopy was deferred, and the haemoptysis resolved spontaneously. Serum creatinine was 2.09 mg/dL (eGFR 24 mL/min/1.73 m^2^, CKD‐EPI 2021), risen from 0.46 mg/dL (eGFR 100) 6 weeks earlier, and reached a nadir of 2.19 mg/dL (eGFR 23) on hospital Day 3; urine output remained preserved. Indirect immunofluorescence showed a perinuclear pattern and antigen‐specific ELISA confirmed MPO‐ANCA at 250.2 U/mL (reference < 1.0 U/mL) with PR3‐ANCA negative. Peripheral eosinophil count was 0%. Urinalysis showed 2+ proteinuria with dysmorphic erythrocytes and no casts; anti‐glomerular basement membrane and anti‐double‐stranded DNA antibodies were negative with normal complement.

**FIGURE 1:**
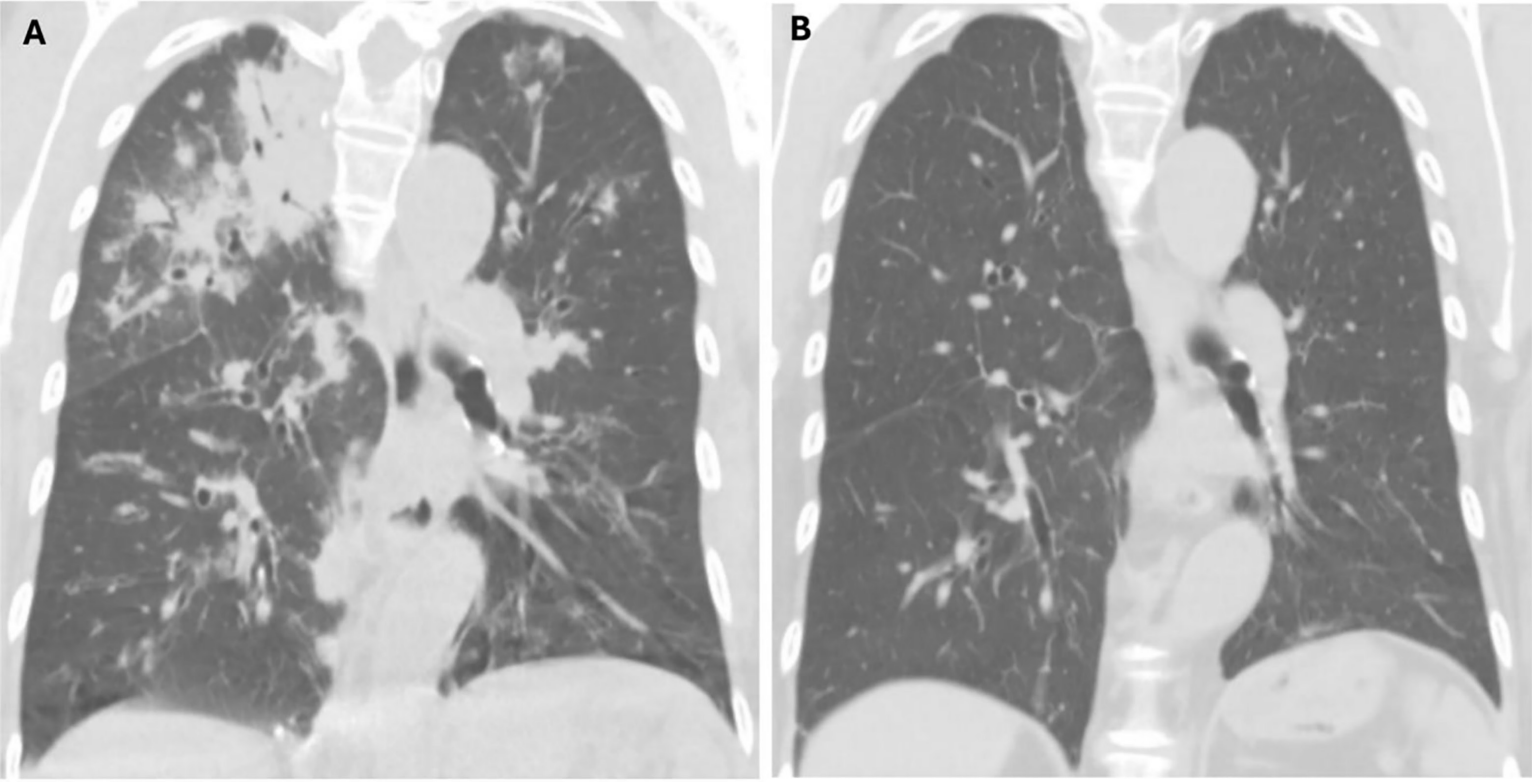
Radiographic response of the pulmonary manifestations to induction immunosuppression. (A) 2025, index presentation, coronal reformatted high‐resolution CT: Multifocal bilateral consolidations and airspace opacities, most confluent in the right upper and middle lobes, superimposed on established bronchial wall thickening and bronchiectasis with scattered mucus plugging. (B) Five months after induction with intravenous methylprednisolone and rituximab, matched coronal reformat: near‐complete resolution of the bilateral consolidations and airspace opacities. The underlying bronchiectasis and bronchial wall thickening persist, consistent with resolution of active vasculitic inflammation superimposed on fixed structural airway disease.

Renal biopsy sampled 14 glomeruli, showing pauci‐immune crescentic glomerulonephritis with a cellular crescent in one glomerulus, no immune deposits and more than half of glomeruli normal—Berden focal class [2]—establishing MPA [3]. There was no comment on eosoniphilic infiltration. Skin biopsy of the purpuric lesions was not pursued as the renal histology already confirmed the diagnosis. Induction comprised intravenous methylprednisolone 500 mg daily for 3 days then an oral prednisone taper, with rituximab 1 g repeated after 2 weeks and 1 g every 6 months as maintenance; Pneumocystis prophylaxis and acid suppression were given. Renal function improved to a peak eGFR of 74 at 2 months and stabilized at 60 mL/min/1.73 m^2^ at 15 months. MPO‐ANCA fell from 250.2 to 5.2 U/mL by 12 months with documented B‐cell depletion, although indirect immunofluorescence remained weakly positive. HRCT at 5 months showed near‐complete resolution of the consolidations and airspace opacities, while the underlying bronchiectasis persisted (Figure 1B). No infusion reactions, infections, or corticosteroid complications occurred. She remains asymptomatic at 15 months (Table 1).

At follow‐up she has been doing well, with resolution of her cough; she has remained adherent to her immunosuppressive regimen and to rheumatology and pulmonary follow‐up.

## 3 Discussion

We reviewed the published literature on bronchiectasis in AAV. Searching PubMed and Europe PMC for bronchiectasis with ANCA‐associated vasculitis, microscopic polyangiitis or MPO‐ANCA, we identified one systematic review with meta‐analysis, two dedicated cohort studies and one bronchiectasis screening study addressing the association, together with three current guideline documents and the ACR/EULAR classification criteria for MPA and EGPA; findings are summarized in Table 2.

**TABLE 2:** Summary of the reviewed literature on bronchiectasis in ANCA‐associated vasculitis and of current guideline positions on etiological testing.

Study	Design	Principal finding relevant to this case
Gu et al. [4] (2024)	Systematic review and meta‐analysis, 24 studies	Pooled bronchiectasis prevalence 19% (95% CI 13%–27%) in AAV; higher with MPO‐ANCA than PR3‐ANCA (28% versus 13%); bronchiectasis may precede, coincide with or follow AAV
Néel et al. [5] (2018)	Retrospective cohort, 58 patients with AAV	Bronchiectasis in 22 patients (37.9%), all MPO‐ANCA positive; independently associated with MPO‐ANCA, female sex and age at diagnosis. Among MPO‐ANCA patients, those with bronchiectasis had more peripheral nerve and less renal involvement
Lhote et al. [6] (2020)	Retrospective multicentre cohort, 61 patients with AAV and bronchiectasis	Bronchiectasis preceded AAV in 25 patients (41%), median 16 years (IQR 4–54); this group had more MPA, MPO‐ANCA and shorter survival
Stainer et al. [7] (2025)	Observational study, standardized autoimmune screening of 468 patients with bronchiectasis	ANCA positive in 3.8%; connective tissue disease diagnosed in 5.6% overall. Authors conclude autoimmune screening should be individualized and prescribed selectively for patients with coexisting features of arthritis, connective tissue disease or systemic vasculitis
Chalmers et al. [8] (2025)	ERS clinical practice guideline	Strong recommendation to test for the underlying cause; ANCA appears only within the etiological testing algorithm, gated behind systemic features, and the guideline states these algorithm components are not separate recommendations
Thomson et al. [9] (2026)	CHEST clinical practice guideline	Addresses management only—13 conditional recommendations on antibiotics, airway clearance, haemoptysis and surgery. Etiological evaluation is outside its scope
Chung et al. [10] (2021)	ACR/VF management guideline	Rituximab‐based induction recommended for severe MPA; bronchiectasis is not addressed as a presenting feature
Suppiah et al. [3] (2022)	ACR/EULAR classification criteria	MPA classification requires ANCA positivity with compatible clinical features; airway disease is not a criterion
Grayson et al. [11] (2022)	ACR/EULAR classification criteria	EGPA classification requires a cumulative score ≥ 6; maximum eosinophil count ≥ 1 × 10^9^/L is the most heavily weighted item (+5). This patient scored 2, excluding EGPA

Abbreviations: AAV, ANCA‐associated vasculitis; ACR, American College of Rheumatology; ANCA, antineutrophil cytoplasmic antibody; CI, confidence interval; ERS, European Respiratory Society; EULAR, European Alliance of Associations for Rheumatology; IQR, interquartile range; MPA, microscopic polyangiitis; MPO, myeloperoxidase; PR3, proteinase 3; VF, Vasculitis Foundation.

Bronchiectasis is common in AAV: a meta‐analysis of 24 studies found a pooled prevalence of 19% (95% CI 13%–27%), higher with MPO‐ANCA than PR3‐ANCA (28% versus 13%) and associated with female sex, peripheral neuropathy and MPA [4]; one cohort reported 37.9% [5]. Less appreciated is the sequence: bronchiectasis preceded AAV in 25 of 61 patients (median interval 16 years), and this bronchiectasis‐first group carried a fivefold higher mortality [6]. Our patient fits that phenotype—female sex, MPO specificity, MPA, and airway disease antedating systemic vasculitis by 7 years.

Whether earlier serology would have altered the outcome is uncertain; two observations argue against a simple causal chain: where bronchiectasis was universally MPO‐associated, affected patients had less frequent renal involvement than those without it [5]; and bronchiectasis in AAV generally does not respond to immunosuppression [4]—consistent with our patient, in whom the consolidations resolved while the established bronchiectasis persisted. The narrower claim stands: 7 years elapsed during which an inexpensive test that would have reframed the diagnosis was never sent.

Current guidance is more nuanced than either endorsement or omission. The 2025 European Respiratory Society guideline strongly recommends standardized testing for the underlying cause of bronchiectasis, but ANCA appears only within its etiological algorithm—an autoimmune panel reserved for patients with systemic symptoms, coexisting autoimmune disease, or haemoptysis or signs of vasculitis—and the guideline states that these algorithm components do not constitute separate recommendations [8]. Systematic screening of 468 bronchiectasis patients found ANCA positivity in 3.8% and concluded screening should remain individualized [7]. A 2026 American College of Chest Physicians guideline addresses management rather than etiological evaluation [9]. Our patient had none of these trigger features before 2025; the gap she illustrates therefore lies outside current algorithms—culture‐negative, radiographically progressive, incompletely corticosteroid‐responsive disease without systemic features.

The combination of MPO‐ANCA positivity, long‐standing airway disease and a history of asthma raises EGPA as the principal alternative diagnosis. Classification criteria are not intended for diagnosis, but applying the 2022 ACR/EULAR criteria is instructive: her cumulative score was 2 points—obstructive airway disease +3, credited on the basis of her asthma history despite normal spirometry, and hematuria −1—well below the ≥ 6 points required, because the most heavily weighted item, a maximum eosinophil count ≥ 1 × 10^9^/L, was never met [11]. Eosinophils were 0% at presentation and had not been elevated on any prior complete blood count; total serum IgE, measured in 2024, was 114 kU/L, without the marked elevation characteristic of EGPA and the renal biopsy did not comment on eosinophilic inflammation. Her asthma was intermittent and non‐progressive with normal spirometry. EGPA is therefore not supported; pauci‐immune crescentic glomerulonephritis with markedly elevated MPO‐ANCA and no tissue or blood eosinophilia establishes MPA.

Renal outcome matched the Berden focal class, which carries the most favourable prognosis and is validated for 1‐ and 5‐year outcomes [2]. The biphasic course—eGFR 74 at 2 months, then stabilization at 60—suggests inflammatory suppression followed by a plateau reflecting residual nephron loss. Discordance between falling MPO‐ANCA titers and persistent immunofluorescence positivity is compatible with non‐MPO perinuclear antibodies during B‐cell depletion, supporting guidance against basing treatment decisions on titers alone [10]. Limitations include unconfirmed alveolar haemorrhage and absent ANCA testing during the airway phase, leaving seroconversion timing unknown.

## Author Contributions

**Andrew Kim:** conceptualization, investigation, writing – original draft, methodology, validation, data curation, writing – review and editing, visualization, supervision. **Celine Vartany:** writing – review and editing, investigation, writing – original draft. **Siddhanth Hegde:** data curation, writing – review and editing, writing – original draft. **Kristie Lau:** writing – review and editing, data curation, writing – original draft. **Lynette Tatosyan:** writing – review and editing, conceptualization, investigation, methodology, supervision, writing – original draft. **Brannen Liang:** conceptualization, investigation, methodology, writing – review and editing, supervision, writing – original draft.

## Funding

The authors have nothing to report.

## Consent

The authors declare that written informed consent was obtained for the publication of this manuscript and accompanying images using the form provided by the Journal. The signed consent form is retained in the authors' records.

## Conflicts of Interest

The authors declare no conflicts of interest.

## Data Availability

The data that support the findings of this study are available from the corresponding author upon reasonable request.
